# Mutations in *PIGY*: expanding the phenotype of inherited glycosylphosphatidylinositol deficiencies

**DOI:** 10.1093/hmg/ddv331

**Published:** 2015-08-20

**Authors:** Biljana Ilkovski, Alistair T. Pagnamenta, Gina L. O'Grady, Taroh Kinoshita, Malcolm F. Howard, Monkol Lek, Brett Thomas, Anne Turner, John Christodoulou, David Sillence, Samantha J.L. Knight, Niko Popitsch, David A. Keays, Consuelo Anzilotti, Anne Goriely, Leigh B. Waddell, Fabienne Brilot, Kathryn N. North, Noriyuki Kanzawa, Daniel G. Macarthur, Jenny C. Taylor, Usha Kini, Yoshiko Murakami, Nigel F. Clarke

**Affiliations:** 1 Institute for Neuroscience and Muscle Research,; 2 Western SydneyGenetics Program, Children's Hospital at Westmead, Westmead, NSW, Australia,; 3 National Institute for Health Research Biomedical Research Centre, Wellcome Trust Centre for Human Genetics, University of Oxford,; 4 The Henry Wellcome Building for Molecular Physiology, University of Oxford, Oxford OX3 7BN, UK,; 5 Discipline of Paediatrics and Child Health, University of Sydney, Sydney, NSW, Australia,; 6 Department of Immunoregulation, Research Institute for Microbial Diseases, and WPI Immunology Frontier Research Center, Osaka University, 3-1 Yamadaoka, Suita, Osaka 565-0871, Japan,; 7 Analytic and Translational Genetics Unit, Massachusetts General Hospital, Boston, MA, USA,; 8 Broad Institute of Harvard and Massachusetts Institute of Technology, Cambridge, MA, USA,; 9 Department of Clinical Genetics, Sydney Children's Hospital, Sydney, NSW, Australia,; 10 Discipline of Genetic Medicine, Children's Hospital at Westmead Clinical School, Sydney, NSW, Australia,; 11 Institute of Molecular Pathology, Vienna 1030, Austria,; 12 Weatherall Institute of Molecular Medicine, University of Oxford, Oxford OX3 9DS, UK,; 13 Murdoch Children's Research Institute, The Royal Children's Hospital, Flemington Road, Parkville, VIC, Australia and; 14 Department of Clinical Genetics, Oxford University Hospitals NHS Trust, Oxford OX3 9DU, UK

## Abstract

Glycosylphosphatidylinositol (GPI)-anchored proteins are ubiquitously expressed in the human body and are important for various functions at the cell surface. Mutations in many GPI biosynthesis genes have been described to date in patients with multi-system disease and together these constitute a subtype of congenital disorders of glycosylation. We used whole exome sequencing in two families to investigate the genetic basis of disease and used RNA and cellular studies to investigate the functional consequences of sequence variants in the *PIGY* gene. Two families with different phenotypes had homozygous recessive sequence variants in the GPI biosynthesis gene *PIGY*. Two sisters with c.137T>C (p.Leu46Pro) *PIGY* variants had multi-system disease including dysmorphism, seizures, severe developmental delay, cataracts and early death. There were significantly reduced levels of GPI-anchored proteins (CD55 and CD59) on the surface of patient-derived skin fibroblasts (∼20–50% compared with controls). In a second, consanguineous family, two siblings had moderate development delay and microcephaly. A homozygous *PIGY* promoter variant (c.-540G>A) was detected within a 7.7 Mb region of autozygosity. This variant was predicted to disrupt a SP1 consensus binding site and was shown to be associated with reduced gene expression. Mutations in *PIGY* can occur in coding and non-coding regions of the gene and cause variable phenotypes. This article contributes to understanding of the range of disease phenotypes and disease genes associated with deficiencies of the GPI-anchor biosynthesis pathway and also serves to highlight the potential importance of analysing variants detected in 5′-UTR regions despite their typically low coverage in exome data.

## Introduction

Glycosylphosphatidylinositol (GPI) acts as a membrane anchor for many cell-surface proteins including receptors, enzymes and adhesion molecules. At least 25 genes are involved in the step-wise production of mature GPI-anchored proteins (GPI-APs) (1). GPI biosynthesis is initiated in the endoplasmic reticulum from phosphatidylinositol (PI) in a reaction mediated by the GPI-*N*-acetylglucosaminyltransferase (GPI-GnT) complex. The GPI-GnT enzyme complex consists of seven proteins including PIG-A, PIG-C, PIG-H, PIG-P, PIG-Q, DPM2 and PIG-Y. Phosphatidylinositol glycan class Y (PIG-Y), the smallest subunit of this complex, is a 71-amino acid protein containing two putative transmembrane domains and it directly associates with the catalytic subunit PIG-A. In the absence of PIG-Y activity, the cell surface levels of GPI-APs are severely decreased (2).

The first disorders to be described in association with deficiencies of the GPI-anchor biosynthesis pathway were paroxysmal nocturnal haemoglobinuria (PNH), caused by somatic mutations in PIGA (3), in haemopoietic cells, and inherited GPI deficiency caused by a hypomorphic promoter mutation in PIGM (4). Whole exome sequencing (WES) technology has facilitated the discovery of pathogenic mutations in several other GPI biosynthesis genes. Germline mutations in PIGL (5), *PIGM* (4), *PIGN* (6), PIGO (7), PIGT (8), PIGV (9), *PIGW* (10), PIGQ (11) and PIGA (12) have all been reported in a small number of families in association with multi-system disease. Common clinical features include moderate to severe developmental delay, seizures and dysmorphic facial features. Reduced levels of GPI-APs CD59, or membrane attack complex-inhibitory protein, and FLAER (fluorescently labelled inactive toxin aerolysin which binds to GPI-linked structures) at the cell surface have been described in many of these patients (13).

We have identified two different sequence variants in *PIGY* in two unrelated families associated with different phenotypes. In Family A, we identified a homozygous c.137T>C variant in two affected sisters presenting with a multi-system disease encompassing dysmorphism, developmental delay, seizures, cataracts and gastrointestinal dysmotility. Levels of two GPI-APs on patient fibroblasts were reduced, providing evidence that these sequence variants impair GPI synthesis and are pathogenic. In Family B, we identified a c.-540G>A mutation in the promoter region in a consensus SP1 site. The affected siblings from Family B have primary microcephaly, moderate developmental delay and are more mildly affected in comparison to Family A. This report identifies *PIGY* as a new disease-causing gene with variable expressivity and increases our knowledge of the phenotypes that can arise from abnormal GPI biosynthesis.

## Results

### Clinical descriptions

#### Family A

**Figure 1.**
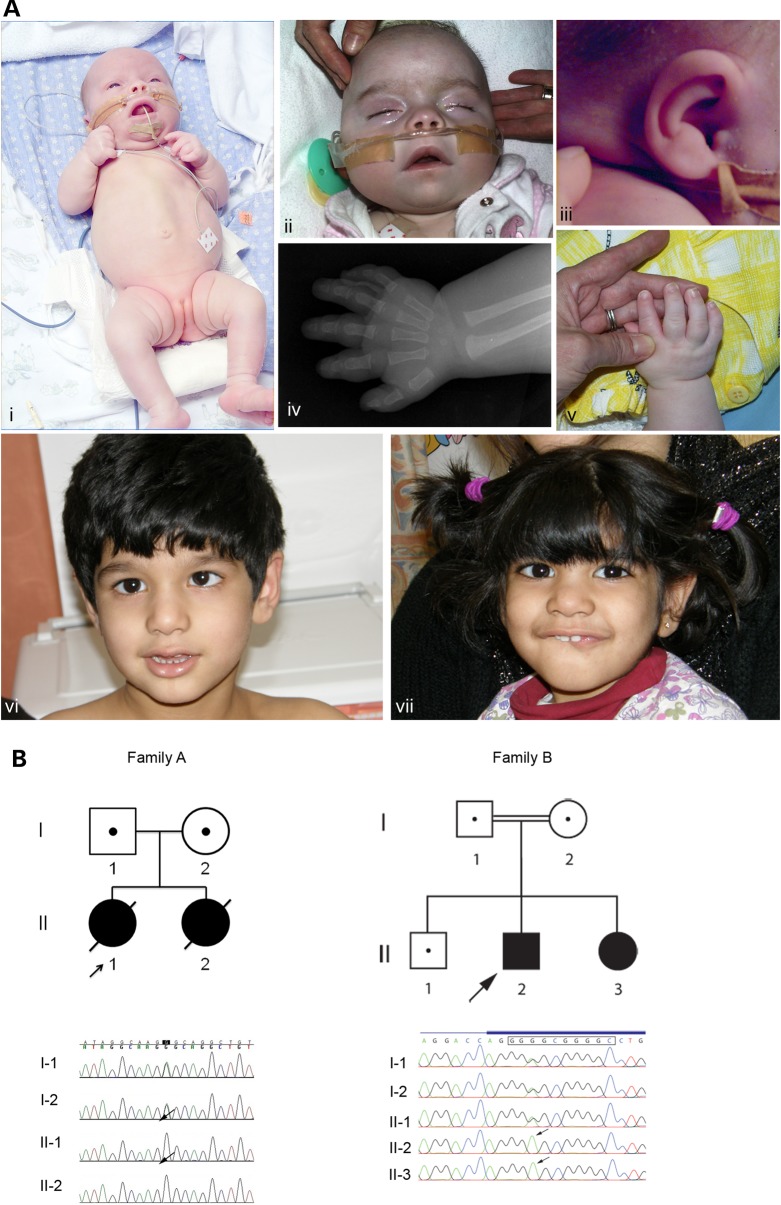
(**A**) Clinical features of patients with mutations in PIGY. Family A, Patient II-1 had proximal limb shortening, left hip dysplasia and contractures were present at the elbows, hips and knees (i). Dysmorphic features included bitemporal narrowing, upturned nares, depressed nasal bridge, deep-set eyes, a short neck (ii) and ears with thickened helices and fleshy earlobes (iii). Brachyphalange, fifth finger clinodactyly and adducted thumbs were present (v). Brachytelephalange was present on X-ray (iv). Family B Patient II-2 (v) and Patient II-3 (vi) had a milder phenotype, with soft dysmorphic features including long palpebral fissures, a bulbous tip to the nose and a wide mouth. Patient II-2 had a strabismus. (**B**) Pedigrees of Families A and B. Shading indicates affected individuals. The probands are indicated with an arrow. Proven heterozygote carriers are shown by a dot. Sanger sequencing validation from Family A confirms the homozygous missense variant c.137T>C (p.Leu46Pro) in the *PIGY* gene (NM_001042616.1) in the two affected sisters. Region shown corresponds to chr4: 89,442,795–813 (hg19). For Family B, Sanger validation and segregation testing of the *PIGY* promoter variant c.-540G>A is shown. The consensus SP1 binding motif is indicated with a black rectangle. Region shown corresponds to chr4: 89,444,938–958.

Two affected sisters were the only children of a non-consanguineous Australian couple of Caucasian decent. Patient II-1 from Family A was born at 32 weeks gestation and had a complicated course with necrotizing enterocolitis and chronic lung disease. She was dysmorphic and had brachyphalangy (Fig. 1), 2–3 toe syndactyly, elbow and knee flexion contractures and severe bilateral hip dysplasia. At 5 months of age, she developed an intractable seizure disorder with multifocal spike and slow wave activity on EEG. This was followed by developmental regression and death at 2 years of age from a respiratory infection.

II-2 had abnormal antenatal scans from 25 weeks gestation with enlarged echogenic kidneys and bowel and long-bone growth failure. Delivery was induced at 28 weeks gestation due to polyhydramnios. Similar dysmorphic features, brachyphalangy, proximal limb shortening, contractures and left hip dysplasia were present. She had bilateral inguinal hernias and bilateral dilatation of the renal collecting systems, with markedly increased echogenicity of the renal parenchyma. Intractable seizures commenced at 6 weeks of age, her development regressed and at 5 months she was mostly unresponsive with poor vision. She died at 7 months of age secondary to an aspiration.

In addition, both affected sisters from Family A had congenital cataracts, intermittent episodes of abdominal distress and vomiting and growth failure. Head growth was normal. MRI brain was normal in both neonates, but showed loss of white matter and cerebral volume in Patient II-1 at 9 months. No abnormalities were present on diffusion-weighted images. Skeletal survey showed osteopenia and brachytelephalangy. Creatine kinase (CK) measurements were persistently elevated in both siblings (554–3640 U/l, normal range = 15–180 U/l). Muscle biopsies were taken from rectus abdominus (II-1, 9 months) and vastus lateralis (II-2, 8 days) showed moderate variation in fibre size with many small, rounded atrophic fibres, increased fibrosis and adipose tissue. No degenerating or regenerating fibres were seen, but in Patient II-2, acid phosphatase staining was increased suggestive of active degeneration.

Alkaline phosphatase levels were elevated in both patients (443–853 u/l, normal range = 40–300 U/l). Extensive metabolic work-up was normal including urine metabolic and oligosaccharide screen, lactate, thyroid function, very long chain fatty acids, 7-dehydrocholesterol, 8-dehydrocholesterol and 8(9) cholestanol, serum transferrin isoforms, white blood cell enzyme testing for CDG type 1A and 1B, white blood cell lysosomal enzymes, bile alcohol analysis, sterol analysis, urine and plasma creatine and guanidine-acetate and CSF lactate, amino acids and neurotransmitters. Neuraminidase on cultured skin fibroblasts were normal. Respiratory chain enzymology was normal in liver. Complex 1-IV were all low relative to protein in muscle, but citrate synthase was also low likely secondary to poor handling and storage. Karyotype was also normal. Additional studies in II-2 included mildly elevated erythrocyte plasmalogen levels and normal serum phytanic acid, normal DHAP-AT and DHAP-s enzyme activity and subtelomere FISH.

#### Family B

Two siblings, born to consanguineous parents of Pakistani descent, were referred with global developmental delay and microcephaly. II-2 was born following an uncomplicated pregnancy and delivery, but had microcephaly at 6 weeks of age. At 5 years this was 46 cm (−3 to −4 SD). His developmental milestones were delayed. He sat with support at 15 months and walked at 3 years of age. His speech was delayed with the acquisition of only short two to three word sentences at the age of 5 years. He was short-sighted with a strabismus and had behavioural difficulties with aggressive outbursts.

II-3 was born following an uncomplicated pregnancy and delivery. She had microcephaly noted at 2 weeks of age and at 2 years had a head circumference of 42.1 cm (−4 to −5SD). Her development was delayed. She sat with support at 18 months and was not walking at the age of 3 years. Her speech was better than her brother's with 10–15 single words at age 3 years. She had poor concentration and was hyperactive.

The parental head circumferences were within the normal range. Neither child had seizures. There was mild facial dysmorphism with long palpebral fissures, a bulbous tip to the nose and a wide mouth (Fig. 1A). Brachytelephalangy was not present.

### Genetic analysis

WES of DNA from II-1 and II-2 from Family A identified a homozygous missense variant c.137T>C (p.Leu46Pro) in the coding region of the *PIGY* gene in both individuals (NM_001042616.1). Sanger sequencing confirmed variant segregation was consistent with an autosomal recessive inheritance pattern (Fig. 1C). Analysis of allelic ratios across chromosome 4 indicated that the homozygous c.137T>C variant lay within a ∼10 Mb segment of autozygosity, suggesting that the parents may be distantly related (Supplementary Material, Fig. S1). *In silico* analysis predicted the variant to be pathogenic (*PolyPhen-2 score of 1.0*) (14) and the variant was not present in the NHLBI Exome Variant Server (http://evs.gs.washington.edu/EVS/, accessed April 2014), 63 000 exomes ExAc (Exome Aggregation Consortium (ExAC), Cambridge, MA, URL: http://exac.broadinstitute.org/, accessed, January 2015) or in the 1000 genomes project (15,16).

For Family B, an autozygosity mapping approach identified candidate regions on 4q22.1-q22.3 and 10p14-pter, both 7.7 Mb in size. WES was performed for the proband (II-2), and the resulting variants were intersected with these two candidate loci. This revealed a c.-540G>A variant in a conserved region of the *PIGY* 5′-UTR. This variant was not in either of the publically available databases mentioned above and was not detected in 274 in-house genomes of mixed ancestry (see www.well.ox.ac.uk/wgs500) or 108 Punjabi individuals from Lahore. However, it should be noted that the region of the variant in Family B is not covered well by either the SureSelect or TrueSeq platforms (Supplementary Material, Fig. S2) and is not covered in the ExAC dataset. Sanger sequencing confirmed segregation consistent with autosomal recessive inheritance (Fig. 1C). Analysis of the exome outside of the homozygous region revealed no likely causative coding variants.

### Patients with p.Leu46Pro substitution in PIG-Y have reduced cell expression of GPI-APs CD55 and CD59 on cultured fibroblasts

**Figure 2.**
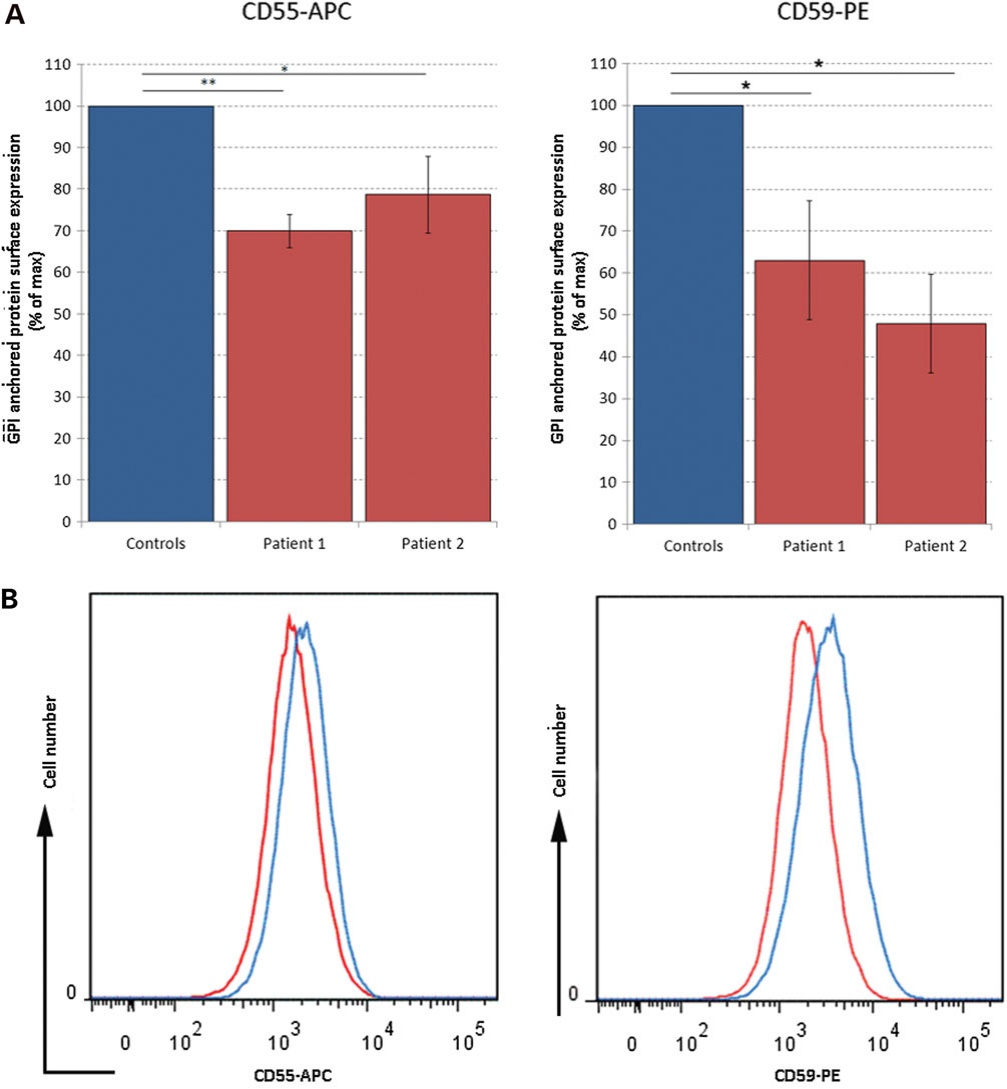
(**A**) Analysis of patient tissue by flow cytometric analysis. Cultured skin fibroblasts from Patients II-1 and II-2 from Family A were labelled with anti-Human CD55-APC and anti-Human CD59-PE and analysed by flow cytometry. (A) Histograms showing the mean fluorescent intensity (MFI) are depicted for Patient II-1 and show a reduction in MFI for CD55-APC and CD59-PE (red) compared with control (blue). (**B**) Graphical representation of these data shows a statistically significant reduction in MFI for these GPI markers. Skin fibroblast biopsies were taken from Patient II-1 and Patient II-2 at 5 months and 8 days, respectively. Primary human fibroblast controls were used from three different individuals at ages 5.5 years, 6 months and 3.5 months. All experiments were performed in triplicate.

Given the known function of PIG-Y, we investigated the effects of the p.Leu46Pro substitution on the surface expression of two GPI-APs, CD55 and CD59. Skin fibroblasts derived from skin biopsies from both affected sisters had a ∼20–50% reduction of cell surface expression of CD55 and CD59 compared with three different controls (Fig. 2A) [For CD55-APC: Patient 1, *P* = 0.0055 (mean fluorescence intensity (MFI) = 2809 ± 521), Patient 2, *P* = 0.0449 (MFI = 2393 ± 582) (pooled controls MFI = 3042 ± 485); for CD59-PE: Patient 1, *P* = 0.0165 (MFI = 2422 ± 921), Patient 2, *P* = 0.0164 (MFI = 1927 ± 582) (Pooled control, MFI = 3988 ± 485) using a standard Students' *t*-test]. These data suggest that the PIGY p.Leu46Pro substitution disrupts GPI biosynthesis or interferes with GPI anchoring capacity, associated with reduced expression of CD55 and CD59 on patient cells. Skin fibroblasts were not available from Family B.

### Transfection of p.Leu46Pro PIG-Y partially rescues PIG-Y deficient cell line

**Figure 3.**
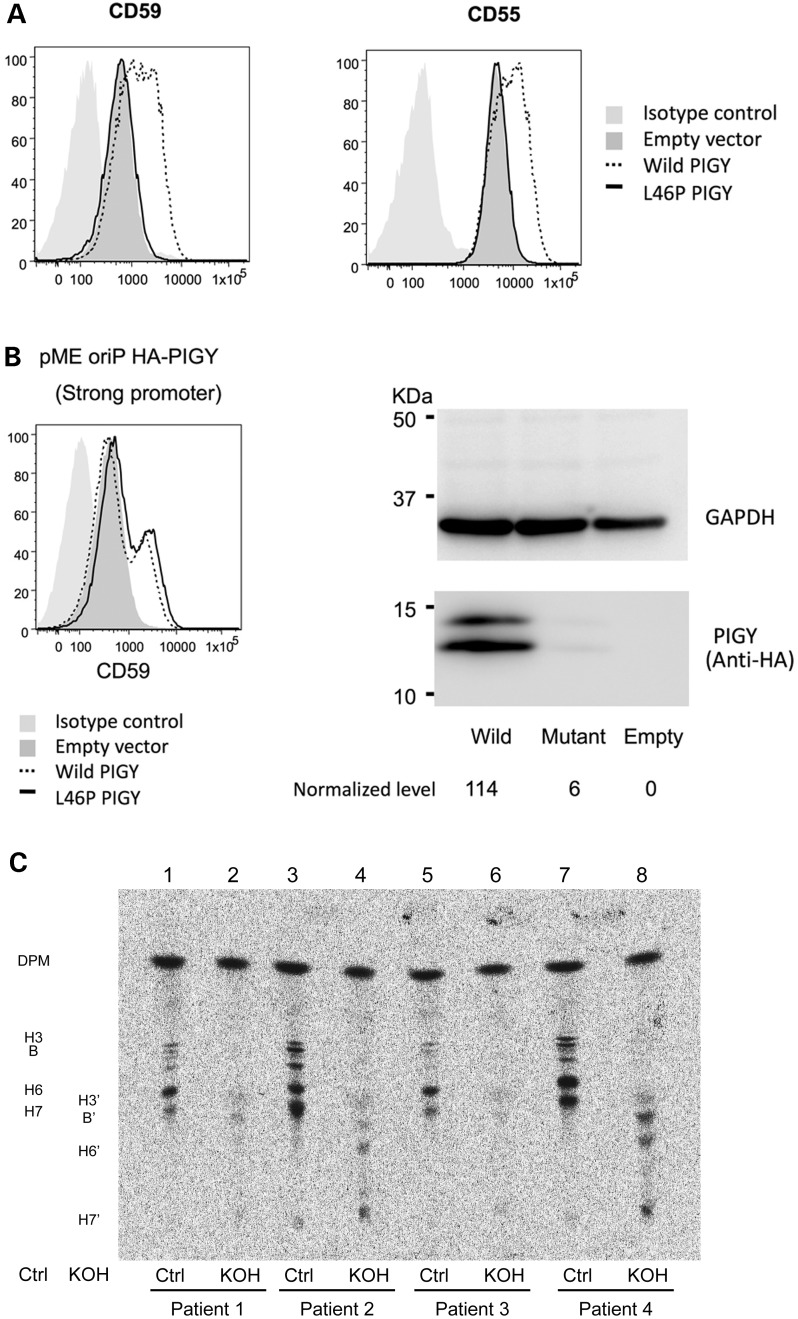
(**A**) Daudi cells (a PIG-Y deficient human Burkitt's lymphoma cell line) were transiently transfected with normal (WT) or mutant (p.Leu46Pro) PIGY driven by minimum TATA box promoter in pTAL oriP puro *PIGY* (weak promoter). Restoration of the surface expression of CD59 and CD55 was assessed 2 days later by flow cytometry. WT-PIGY restored the surface expression of CD55 and CD59, but p.Leu46Pro mutant PIG-Y did not. (**B**) After transient transfection of Daudi cells with normal (WT) or mutant (p.Leu46Pro) *PIGY* driven by the strong promoter in pME-oriP HA-PIGY, restoration of surface expression of CD59 was achieved by WT and p.Leu46Pro constructs, indicating that the mutant has some residual activity (left panel). Cell lysates prepared from Daudi cells transfected with either WT-PIGY, mutant p.Leu46Pro *PIGY*, and empty vector were separated by SDS-PAGE and probed for anti-HA and anti-GAPDH antibodies. Normalization of the anti-HA signal compared with GAPDH showed a marked reduction in protein expression in the mutant (p.Leu46Pro) compared with WT. HA-tagged PIG-Y appeared as doublet bands on western blotting for an unknown reason. (**C**) *In vivo* labelling of ^3^H-mannose into transformed fibroblast cells derived from Patients II-1 and II-2 (Family A) was performed to enhance incorporation into GPI mannolipids. Fibroblasts derived from Patient 2 (Family A) had KOH-resistant alkyl-bond-containing GPI spots similar to fibroblasts from a healthy control 1 (spots in KOH lanes). Fibroblasts from Patient 1 and another healthy control were not efficiently labelled by mannose. DPM, dolichol-phosphate mannose; H3, B, H6 and H7, mannose-containing GPI precursors; H3′, B7, H6′ and H7′, GPI precursors converted from H3, B, H6 and H7, respectively, by KOH-treatment.

We permanently transfected Daudi cells (PIG-Y-deficient human Burkitt's lymphoma cell line) with wild-type (WT) or p.Leu46Pro mutant vector constructs driven by the weak promoter followed by assessment of cell surface expression of CD55 and CD59 by FACS analysis. WT-PIG-Y transfection restored the cell surface expression of both CD55 and CD59, but the p.Leu46Pro mutant PIG-Y did not (Fig. 3A). Levels of their expression by the mutant PIGY were similar to the empty vector control. To test if the lack of activity is due to the instability of the mutant PIG-Y protein, stronger (17) expression was needed for detection by western blotting. For this, Daudi cells were transiently transfected with WT or mutant (p.Leu46Pro) pME-oriP HA-PIGY vectors. With this strong promoter-driven vector, the mutant *PIGY* restored the surface expression of CD59 similarly to WT *PIGY*, indicating that the mutant PIG-Y likely has some GPI biosynthetic activity (Fig. 3B). As demonstrated by western blotting, the mutant protein expression was significantly decreased compared with when WT-PIGY was transfected (Fig. 3B). These data suggest that the p.Leu46Pro mutation reduces PIG-Y protein stability leading to lower protein levels and a reduced capacity to synthesize GPI anchors for correct protein targeting to the cell surface.

### Patients with p.Leu46Pro substitution in PIG-Y have normal GPI lipid remodelling

Radiolabelling of tritiated mannose into tunicamycin-treated transformed patient fibroblast cells was performed to investigate whether GPI protein anchors in Family A were abnormal in their structure as well as their quantity, as occurs in peroxisomal disorders. GPI precursors in fibroblasts derived from Patients 1 and 2 (Family A) had KOH-resistant alkyl bonds similar to that observed in similarly transformed control fibroblasts (Fig. 3C, bands in KOH lanes). These data suggest that patient cells harbouring the p.Leu46Pro *PIGY* mutation can remodel GPI lipid to the alkyl–acyl form.

### Effect of c.-540G>A variant on gene expression and GPI anchor biosynthesis

**Figure 4.**
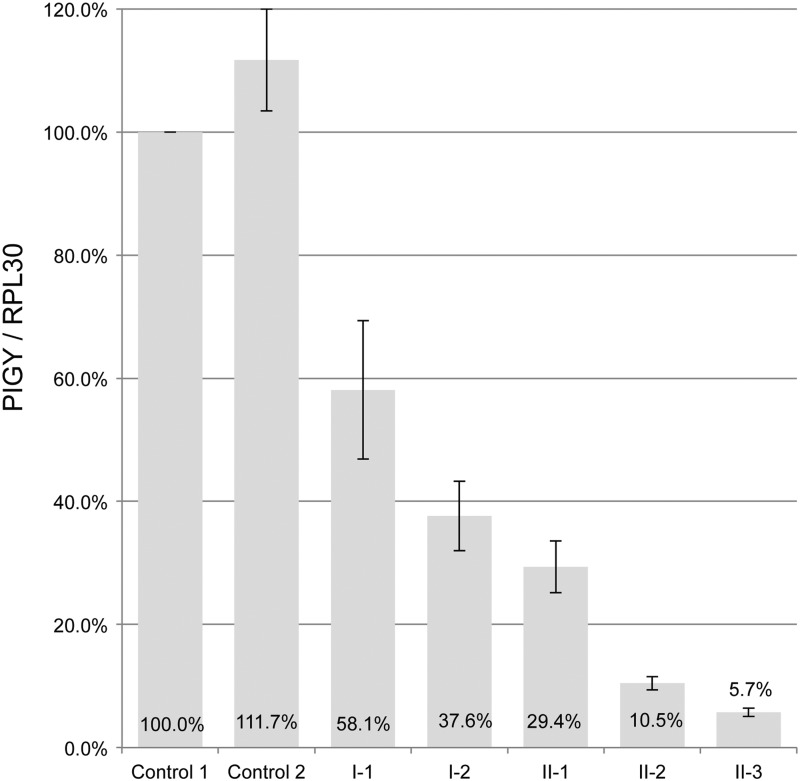
Expression analysis in family B using qPCR. Expression analysis was performed using RPL30 as a control gene, and the results are normalized with respect to the first control individual. qPCRs were performed in triplicate and then the experiment was repeated three times. Error bars represent the standard deviations obtained across the three runs. The RNA used for this experiment was from whole blood samples extracted using the PAXgene blood RNA kit.

As the c.-540G>A variant disrupts a consensus SP1 transcription factor binding site (GG**G**GCGGGGC > GG**A**GCGGGGC) in which there are no other variants reported in dbSNP138, we hypothesized that it might influence *PIGY* gene expression. Sanger sequencing of genomic DNA confirmed that the father (I-1) and the unaffected sibling (II-1) were heterozygous for the A:T haplotype (c.-540G>A:rs3177413) and so could be used to directly compare the relative allelic expression. RT-PCR and Sanger sequencing of two independent blood samples obtained from each of these individuals indicated that expression was consistently higher from the WT allele (data not shown). However, Sanger sequencing does not have a high sensitivity for variant detection when one allele is present at low levels (18). Therefore, to better quantify the relative expression, sequencing was repeated using the Ion Torrent PGM and this showed that only 7–11% of transcripts originate from the mutant allele (Table 1). These results were consistent with qPCR, which showed that for both affected individuals (II-2 and II-3), the *PIGY* expression was at 6–10% compared with an unrelated control (Fig. 4), whereas for the three heterozygote carriers in family B, expression levels were intermediate. Despite this decrease in the *PIGY* transcript levels, we could detect no significant reduction in granulocyte CD16 surface expression for the two individuals homozygous for the c.-540G>A mutation (Supplementary Material, Fig. S3).

**Table 1.** Quantitative analysis of *PIGY* allelic expression levels in Family B using Ion Torrent PGM sequencing

Amplicon details			Sample information and genotype			Sequencing rs3177413 on coding strand (CS1)				Sequencing rs3177413 on non-coding strand (CS2)			
Template	Primer sequences (common sequence CS1 and CS2 tags underlined)	Target size	ID	SP1 mutation (c.-540G>A)	rs3177413 (c.-222C>T)	T	C	Total	Percentage T	T	C	Total	Percentage T
cDNA	CS1-PIGY-1Fv2: 5′-ACACTGACGACATGGTTCTACAGGGGCAAGAAGACTGAGGA-3′; CS2-PIGY-2Rv2: 5′-TACGGTAGCAGAGACTTGGTCTAACTAGCGCTGCTCCACTTC-3′	232 bp	I-1	A/G	T/C	168	1761	1929	**8**.**71**	210	2248	2458	**8**.**54**
			II-1	A/G	T/C	156	1439	1595	**9**.**78**	223	1911	2134	**10**.**45**
			Control 1	G/G	T/C	981	1190	2171	45.19	1110	1432	2542	43.67
			Control 2	G/G	C/C	2	2078	2080	0.10	2	2099	2101	0.10
			I-2	A/G	T/T	2190	5	2195	99.77	2087	3	2090	99.86
cDNA	CS1-PIGY-1Fv3: 5′-ACACTGACGACATGGTTCTACAGACCGGGGCAAGAAGACT-3′; CS2-PIGY-2Rv3: 5′-TACGGTAGCAGAGACTTGGTCTTGTCATCCTAGCTGCCTGTG-3′	195 bp	I-1	A/G	T/C	544	4361	4905	**11**.**09**	277	2238	2515	**11**.**01**
			II-1	A/G	T/C	389	4731	5120	**7**.**60**	160	2072	2232	**7**.**17**
			Control 1	G/G	T/C	1899	2006	3905	48.63	1010	973	1983	50.93
			Control 2	G/G	C/C	9	4787	4796	0.19	1	1387	1388	0.07
			I-2	A/G	T/T	4864	10	4874	99.79	2576	9	2585	99.65
gDNA	CS1-PIGY-intronF: 5′-ACACTGACGACATGGTTCTACATGAAAATCAGGCTCTTCAAGC-3′; CS2-PIGY-2R: 5′-TACGGTAGCAGAGACTTGGTCTCTAGCGCTGCTCCACTTCTT-3′	210 bp	I-1	A/G	T/C	2899	2814	5713	50.74	1988	1625	3613	55.02
			II-1	A/G	T/C	2635	2880	5515	47.78	2047	1802	3849	53.18
			Control 3	G/G	C/C	8	6740	6748	0.12	8	4905	4913	0.16
			I-2	A/G	T/T	5605	12	5617	99.79	3633	3	3636	99.92

Next generation sequencing of cDNA fragments derived from SP1 mutation carriers confirmed that the mutant *PIGY* allele is expressed at lower levels than the WT allele. As the mutation in the SP1 consensus sequence (located at chr4:89,444,948, hg19) is 4 bp from the transcription start site, this position could not be interrogated directly in cDNA. Instead, the rs3177413 C/T polymorphism was used as a surrogate for the SP1 mutation—familial transmission confirmed that the SP1 mutation is *in cis* with the T allele at rs3177413. Based on raw read counts from two independent cDNA templates, the C/T allelic ratio at rs3177413 (located at chr4: 89 443 162) showed that in heterozygote carriers, only 7–11% of RNA was expressed from the mutant allele (in bold). In contrast, a control subject who was heterozygous for rs3177413 but who did not have the SP1 mutation showed biallelic expression (44–51% from T allele). Sequencing of all samples was performed on a single 314 chip using the Ion Torrent PGM using the Ion Torrent variant caller software after deconvolution of individual sample barcodes. Ion Torrent bidirectional sequencing allows the data derived from the coding and non-coding strands to be analysed independently, and these were shown to be in good agreement with one another. Analysis of genomic DNA from heterozygous samples at rs2177413 were added as internal controls to confirm that there was no amplification or sequencing bias towards one allele, while rs3177413 homozygote samples confirmed that sequencing noise at this position was minimal (<0.2%). Variant positions annotated with reference to NM_001042616.1.

## Discussion

We describe two families with homozygous recessive mutations in *PIGY*, establishing *PIGY* as a new disease-causing gene in humans. Two affected sisters from Family A, who were homozygous for a missense mutation in *PIGY* (p.Leu46Pro), died from severe multi-system disease while two affected siblings from Family B, homozygous for a mutation in the promoter region of *PIGY* (c.-540G>A), presented with moderate developmental delay and microcephaly. The p.Leu46Pro substitution involves a conserved residue in the second putative transmembrane domain (2). Amino acids in this domain and the carboxy-terminal cytoplasmic region are relatively more conserved among species and may be important for interaction with PIG-A, a catalytic component of GPI-GnT. The substitution of leucine to proline, which is less hydrophobic and is inhibitory to the formation of α-helices, might affect association with PIG-A, thereby causing instability of mutant PIG-Y protein as suggested by our results (Fig. 3B). Fibroblasts derived from both affected sisters from Family A show a 20–50% reduction in protein expression of CD55 and CD59 at the cell surface by flow cytometric assessment, consistent with a defect in GPI-anchor biosynthesis in these patients. Using a cell culture model, we show that expression of mutant p.Leu46Pro PIG-Y in the PIG-Y deficient Daudi cell line only partially restores the cell surface expression of the GPI protein markers CD55 and CD59. Taken together, these results provide strong evidence that the p.Leu46Pro substitution leads to defects in GPI-APs in patient cells.

Family A had multi-system disease consistent with previously described disorders of GPI anchor biosynthesis, whilst Family B had less severe disease limited to microcephaly and global developmental delay. This is likely due to differences in the mutations between the two families and their effects on PIG-Y protein function. We have shown that the promoter mutation in Family B markedly reduces *PIGY* transcript levels in blood, which likely leads to reduced expression of functionally normal PIG-Y protein. We hypothesize that the level of PIG-Y expression in most tissues in Family B is sufficient for many aspects of health, explaining why Family B's phenotype is more limited. The effects of the SP1-binding site mutation on *PIGY* transcript levels may also vary between tissues. Indeed, initial experiments showed no difference in levels of CD16, a GPI-AP, in granulocytes from affected individuals from Family B, suggesting that PIG-Y levels are sufficient for normal GPI biosynthesis in that tissue. The only known cases of GPI deficiency caused by promoter mutations are PIGM deficiency (4). Two individuals with the same homozygous promoter mutation had hepatic vein thrombosis, which has never been reported in GPI deficiency caused by mutations in protein coding regions. It is therefore not surprising that affected individuals from Family B who had homozygous promoter mutation had phenotypes quite different from those of affected individuals from Family A who had mutations in coding region. In contrast, the mutation in Family A will lead to expression of the p.Leu46Pro mutant PIGY protein in all tissues, which likely accounts for the severe, multi-system involvement.

There is growing recognition that autosomal recessive disorders of GPI biosynthesis are an important subclass of the congenital disorders of glycosylation. Over 150 proteins require GPI anchorage for cell-surface expression and all cell types in the body employ this anchoring mechanism (1). Previously published defects of GPI anchor biosynthesis present in infancy with multi-system disease, likely due to the wide range of biological processes affected by the reduced expression of GPI-APs. The findings in our patients help to understand the range of phenotypes that can arise (Table 2). Common dysmorphic features found in a range of GPI anchorage disorders include bi-temporal narrowing, anteverted nares, depressed nasal bridge, long philtrum, high palate and a tented upper lip. The ears are commonly large with fleshy lobes and overfolded helices. Fifth finger clinodactyly and nail hypoplasia are commonly described. Seizures, moderate-to-severe developmental delay, growth failure and cerebral visual impairment are commonly described. Other common features include genitourinary defects, gastrointestinal dysfunction and cardiac defects (Table 1). The sisters from Family A share many features with other inherited GPI anchor defects but are the first to be described with cataracts. The hyperphosphatasia often observed in inherited GPI deficiency occurred albeit mildly in the two affected individuals from Family A. When mature GPI available to precursor alkaline phosphatase was not sufficient, some alkaline phosphatase is transferred to water instead of GPI, resulting in secretion of soluble enzyme. Recently, it has been reported that pyridoxine may be effective in patients with mutations in PIGO (19) and butyrate may be effective in patients with PIGM (20), raising important therapeutic considerations.

**Table 2.** Comparison with features of described disorders of GPI anchorage

Clinical feature	PIGY Family A (c.137C>T)	PIGY Family B (c.-540G>A)	PIGA (germline mutation)	PIGL	PIGW	PIGM	PIGV	PIGN	PIGO	PIGT
Pregnancy and birth	Polyhydramnios	−						Polyhydramnios		
Dysmorphic features	High forehead, bi-temporal narrowing, upturned nares, depressed nasal bridge, high palate, thick, short neck Fifth finger clinodactyly 2–3 syndactyly	Mildly dysmorphic with long palpebral fissures, bulbous tip to nose, wide mouth, squint	Pierre Robin sequence, depressed nasal bridge, short, anteverted nose, malar flattening, upslanted palpebral fissures, small mouth with downturned corners and a triangular shape, short neck Nail hypoplasia	+	Broad nasal bridge, tented upper lip		Round face, downturned mouth, long palpebral fissures, prominent nasal bridge Nail hypoplasia, hypoplasia of the terminal phalanges Supernumerary nipples	Bi-temporal narrowing, small nose, upturned nares, long philtrum, open mouth, tented upper lip, high palate, micrognathia	Hypertelorism, downward-slanting palpebral fissures, short nose with broad nasal bridge and tip, long philtrum, facial asymmetry, tented mouth Fifth finger clinodactyly Nail hypoplasia Broad distal phalanx of the thumb	High forehead, bi-temporal narrowing, depressed nasal bridge, long philtrum with a deep groove, open mouth
Mild macrosomia and macrocephaly	−	−	+				+	+	−	+
Hypotonia	Truncal hypotonia with brisk reflexes	−	Truncal hypotonia with brisk reflexes	+			+	+		+
Moderate–severe developmental delay	+	+		+	+		+	+	+	+
Growth delay	+	−					+		+	
Seizures	+	−	+	+	Tonic spasms	+	+	+	+	+
Ophthalmologic features	Cataracts cerebral visual impairment	Strabismus		Coloboma			Strabismus	Nystagmus, cerebral visual impairment, wandering eyes	−	Nystagmus, strabismus cerebral visual impairment, hyperopia abnormal eye motility
Ears and hearing		−	Overfolded helix	Conductive hearing loss			Thickened helices	Large ears with fleshy prominent ear lobe, overfolded helix	Large ears with fleshy lobes	Normal hearing
Dental		−	Gingival hypertrophy					Gingival hypertrophy		Premature loss of incisors
Genitourinary tract	Dilated renal collecting systems and increased echogenicity of renal parenchyma	−	Vesicoureteral reflux and a duplicated collecting system		Inguinal hernia		Hydronephrosis	Hydrocele, dilatation of renal collecting system	−	Nephrocalcinosis, ureteral dilatation, unilateral renal cysts and dysplasia
Gastrointestinal	Poor feeding, abdominal distension and vomiting, necrotizing enterocolitis	−					Abdominal distension and vomiting Anterior anus Short-segment Hirschsprung	Feeding and swallowing difficulties. Gastroesophageal reflux. Anal stenosis, imperforate anus, ano-vestibular fistula	Anal stenosis, anal atresia, perineal fistula	
Brain		Small brain	Thin corpus callosum, delayed maturation of white matter, absent septum pellucidum, dilated lateral ventricle, hypoplasia of the cerebellum and vermis					Mild enlargement of ventricular spaces Delayed maturation of white matter and thin corpus callosum. Hypoplasia of the cerebellum and vermis	Enlarged supra-tentorial ventricular system	Variable—frontotemporal atrophy and cerebellar hypoplasia, primitive Sylvian fissures, severe cerebellar atrophy with vermis hypoplasia
Cardiac	–	–	Atrial septal defect, patent ductus arteriosus	Congenital heart disease				Atrial septal defect, peripheral pulmonary stenosis, patent ductus arteriosus	Atrial septal defect, peripheral pulmonary stenosis	Restrictive cardiomyopathy and PDA
Skeletal features	Flexion contractures of the elbows, hip dysplasia. Proximal limb shortening Short fingers and small feet Radiographic—osteopenia, shortening of the small tubular bones of the hands and feet, delayed bone age	–	Craniosynostosis, prominent occiput Hip, knee and elbow contractures. Broad palms with short fingers and poor central tone with brisk reflexes				Brachytelephalangy Mild scoliosis	Tapering fingers, brachydactyly, deep plantar groove between first and second toes, small feet, joint contractures	Brachytelephalangy of digits II–V. Left coronal synostosis	Pectus excavatum, scoliosis, short upper extremities, craniosynostosis Radiographic—bracycephaly, slender and osteopenic long bones with relatively large secondary ossification centres, wide and long femoral necks with enlarged secondary ossification centres, short ulnae and delayed bone age
Haematological		–	Paroxysmal nocturnal haemoglobinuria			Portal and hepatic vein thrombosis		–		
Other				Migratory ichthyosiform dermatosis						Atypical lobulation of the lungs Mild elevated of plasma calcium, hypercalciuria and low PTH
ALP	Elevated	Normal			Elevated	–	Elevated	–	Elevated	Low
References			Johnston *et al.* (12)	Ng *et al.* (5)		Almeida *et al.* (4)	Thompson *et al.* 2011	Maydan *et al.* (6)	Krawitz *et al.* (7)	Kvarnung *et al.* (8)

The two severely affected patients (Family A) in this study share many clinical features in common with rhizomelic chondrodysplasia punctata type 1 (RCDP1), a peroxisomal disorder which also presents with proximal shortening of the long bones, growth retardation, cataracts, severe intellectual disability, seizures and early death. However, the absence of punctate calcification in our patients is inconsistent with this disorder. Plasmalogens were mildly elevated, phytanic acid was normal and DHAP-AT and DHAP-S activity on cultured skin fibroblasts was normal. Lipid remodelling of GPI anchors occurs in a later part of the GPI biosynthetic pathway and is dependent on the alkyl-phospholipid biosynthetic pathway in the peroxisome. Kanzawa *et al.* showed that patients with mutations in genes involved in peroxisomal biogenesis had defective GPI lipid remodelling and were unable to synthesize 1-alkyl-2-acyl GPIs (21). Unlike patients with RCDP1 and Zellweger syndrome, patients with p.Leu46Pro substitution in PIGY do not have defective GPI lipid remodelling and are able synthesize alkyl–acyl GPI forms. Inherited GPI deficiencies are an important differential for peroxisomal disorders and GPI lipid remodelling might be used as a tool to differentiate between these two clinically similar disorders.

In Family B, even though we could restrict the search space to <1% of the genome based on autozygosity, initial filtering of the 217 641 variants detected by WES did not yield any likely causative variants. It was only after extending our definition of ‘deleterious’ to include 5′-UTR variants that lie in regions annotated by the ENCODE consortium (22) as transcription factor binding sites, that we uncovered the c.-540G>A variant. UTR regions were not targeted by first-generation exome capture kits and even when capture probes are present in these regions, sequence coverage is often low due to high GC content (Supplementary Material, Fig. S2). Although variants in these regions are becoming less refractory to detection as researchers switch from exome analysis to WGS, they remain difficult to interpret. Our detection of a functional homozygous 5′-UTR variant, despite only having four good quality reads, thus represents an unanticipated finding that highlights the importance of developing better tools to analyse the non-coding portion of the genome.

In conclusion, we describe the first report of two different recessive mutations in the GPI biosynthesis gene *PIGY* that are associated with different phenotypes. The p.Leu46Pro mutation in the coding region of the *PIGY* causes a severe congenital, multi-system disorder resulting in early death, whilst the promoter mutation identified in Family B is associated with a moderate CNS phenotype compatible with life. This study contributes to understanding of the phenotype of disorders associated with deficiencies of the GPI-anchor glycosylation pathway, an important differential diagnosis in infants with multi-system disease, epileptic encephalopathy, dysmorphic facial features, brachytelephalangy and hyperphosphatasia.

## Material and Methods

### Genetic analysis

This research was approved by the Human Research Ethics Committee of the Children's Hospital at Westmead, Australia (10/CHW/45) and the Wales Research Ethics Committee, UK (12/WA/0001). WES was performed on genomic DNA from two sisters and their parents from Family A in collaboration with the Broad Institute using methods previously reported (23). In Family B, SNP-array-based autozygosity mapping was performed on all close family members using human CytoSNP12v2 (Illumina). WES of the proband (II-2) and data analysis were performed as previously described (24). Segregation of both variants was confirmed by Sanger sequencing.

### Cell culture and flow cytometry studies

All tissue culture media and reagents were purchased from Life Technologies unless otherwise stated. Skin fibroblast cells were grown in a Dulbecco's Modified Eagles Media (DMEM)/F12-HAMS supplemented with 10% foetal bovine serum (FBS) and gentamicin (50 µg/ml) and grown in 5% CO_2_ incubator. To investigate levels of GPI-APs, cultured skin fibroblasts derived from Patient II-1 in Family A (a full thickness skin biopsy taken at 5 months), Patient II-2 in Family A (8 days) and three controls (ages 3.5 months, 5.5 months and 5.5 years) were treated with versene and incubated with allophycocyanin (APC)-conjugated mouse anti-human CD55 and phycoerythrin (PE)-conjugated mouse anti-human CD59 antibodies (BD Biosciences) in phosphate buffered saline (PBS) without calcium or magnesium and supplemented with 2% FBS for 30 min at 4°C. Cells were then centrifuged at 250*g* for 5 min and washed twice in the above solution. Labelled cells were analysed using a CantoII flow cytometer (BD Biosciences) and analysed using FlowJo™ v 7.6.5 software. Experiments were performed in triplicate from each cell line, and the results were averaged. Fibroblasts were not available from members of Family B and so whole blood samples were used instead. These were treated with ACK lysis buffer and stained with anti-human CD16 (Invitrogen); samples were run on a BD FACSCanto and data analysed by FlowJo. Granulocytes were identified according to FSc and SSc profile.

### Functional analysis of mutant PIGY cDNA

Daudi cells (PIG-Y deficient human Burkitt's lymphoma cell line) expressing EBNA1 antigen (2,25) were permanently transfected with WT or c.137T>C mutant PIG-Y constructs containing Epstein–Barr virus oriP. For low level expression, *PIGY* cDNA was driven by minimum TATA box promoter in pTAL oriP puro PIGY. Cells (10^7^) were suspended in 0.8 ml of Opti-MEM and electroporated with 20 µg each of the plasmids at 350 V and 500 µF using a Gene Pulser (Bio-Rad, Hercules, CA). After selection with 0.5 mg/ml of puromycin for 2 weeks, restoration of the surface expression of GPI-APs was determined by staining cells with mouse anti-human CD59 (5H8), human CD55 antibodies and each isotype IgG followed by a PE-conjugated anti-mouse IgG antibody (mouse IgG1 and IgG2a, and secondary antibody were purchased from BD Biosciences) and analysed by flow cytometer (Canto II) using Flowjo software (Tommy Digital Inc., Tokyo, Japan). For high level expression, PIG-Y deficient Daudi cells were transiently transfected with WT or mutant (c.137T>C) strong promoter-driven pME-oriP HA-PIGY. Two days later, restoration of the surface expression of CD59 was assessed by flow cytometry. Lysates were applied to SDS-PAGE, and western blotting was performed using anti-HA antibody (HA-7, Sigma, St. Louis, MO). The levels of protein expression were normalized with the intensities of glyceraldehydephosphate dehydrogenase (GAPDH), the loading control and luciferase activities used for evaluating transfection efficiencies.

### GPI mannolipid analysis

Patient and control fibroblasts were transformed using telomerase gene and SV40 large T gene and metabolically radiolabeled with tritiated mannose in the presence of tunicamycin to enhance incorporation into GPI mannolipids by preventing incorporation into more abundant N-glycan precursors. Radiolabeled GPI mannolipids were treated with 0.1 N KOH to cleave ester linkages and then analysed by thin layer chromatography on silica gel followed by phosphoimaging to detect tritiated GPI (26).

### Gene expression analysis

RNA was extracted from 2.5 ml of blood using the PAXgene kit (Qiagen) and Reverse Transcriptase (RT) reactions were performed using the QuantiTect RT kit (Qiagen). Relative allelic expression of *PIGY* was measured by analysing cDNA using both Sanger sequencing and a 314 chip run on the Ion Torrent PGM platform. As the c.-540G>A variant is only 4 bp from the transcription start site, expression levels had to be measured indirectly using the T allele at rs3177413 (c.-222C>T) which familial transmission showed was *in cis* with c.-540G>A allele. RT-PCR primer sequences are shown in Table 1. The first round of amplification (25–30 cycles) and second round of amplification (15 cycles, with barcoding primers) were both performed with the FastStart Taq DNA polymerase kit (Roche). Samples were pooled, cleaned using Ampure beads, quantified using the BioAnalyser (Agilent) and diluted to 10 pM. Emulsion PCR and sequencing were carried out according to manufacturer instructions.

Quantitative PCR was performed using IQ SYBR Green Supermix (BIO-RAD) and the iQ5 Real-Time PCR Detection System (BIO-RAD) and the following primers: PIGY-V5-F 5′-AGGGATGTTCATCTCCAACCA-3′, PIGY-V5-R 5′-TGCGCATATCAGGCTTAGGA-3′, RPL30-F 5′-CAGACAAGGCAAAGCGAAAT-3′ and RPL30-R 5′-TGGACACCAGTTTTAGCCAAC-3′. PCRs were performed in triplicate, and the experiment was carried out three times.

## Authors contributions

### Sydney, Australia Group

The Sydney Group lead by Nigel Clarke (senior author) identified the gene and lead the collaborative studies to ascribe pathogenicity. Both Nigel Clarke and Kathryn North were responsible for study concept and design, analysis and interpretation of data, study supervision obtaining funding and drafting/revising manuscript for content. Biljana Ilkovski's (co-first author) contribution has been to perform the flow cytometric experiments on the patient cells (Fig. 2), assemble the figures and take a lead role in the preparation of the manuscript. Gina O'Grady (author position #2) identified the gene via WES, confirmed the mutation and family inheritance, reviewed and assembled detailed clinical and pathological information, compiled a detailed clinical table of all known PIG phenotypes, helped write and edit the manuscript. Leigh Waddell was involved in mutation identification and confirmed the mutation by Sanger Sequencing and edited the manuscript. Fabienne Brilot helped direct flow cytometeric experiments and edited the manuscript. David Sillence, Anne Turner and John Christodoulou were the clinicians who described the clinical phenotypes of the patients, contributed to diagnostic investigations and edited the manuscript.

### UK Group

The Oxford Group lead by Usha Kini and Jenny Taylor identified the gene in the UK family. Alistair Pagnamenta (co-first author) performed SNP array experiments, homozygosity mapping, analysed exome data, did RNA extractions, designed and performed RNA sequencing, was involved in the flow cytometry experiments and helped write and edit the manuscript. Malcolm Howard performed the qPCR and Sanger validation of the UK Family mutation, analysed exome data and helped with the flow cytometry. Samantha Knight supervised and reviewed the SNP array data. Consuelo Anzilotti helped perform the flow cytometry experiments. Anne Goriely helped design, perform and interpret data from the PGM RNA sequencing experiment. Niko Popitsch analysed the exome coverage in the two families and compared with in house WGS data. David Keays was involved with setting up of the Oxford Brain Abnormalities group.

### Boston, USA Group

The Boston Group lead by Daniel MacArthur identified the gene in the Australian family via WES. Monkol Lek and Brett Thomas performed the Bioinformatics of WES to ascribe pathogenicity. All authors revised and edited the manuscript.

### Japanese Group

The Japanese Group lead by Taroh Kinoshita lead the collaborative studies to examine the functional consequences of the PIGY mutation in the Australian family using cell culture models lipid remodelling studies, helped write and edit the manuscript. Yoshiko Murakami and Noriyuki Kanzawa were responsible for designing the flow cytometric experiments examining expression of PIG-Y constructs into PIG-Y deficient cells as well as the lipid remodelling studies and helped write and edit the manuscript.

## Supplementary Material

Supplementary Material is available at *HMG* online.

## Funding

This work was supported by the National Health & Medical Research Council of Australia (APP571287—KN, NC, APP1035828—NC and APP1022707—KN, NC) and National Institute for Health Research (NIHR) Biomedical Research Centre Oxford with funding from the Department of Health's NIHR Biomedical Research Centres funding scheme. We thank the High-Throughput Genomics Group at the Wellcome Trust Centre for Human Genetics (funded by Wellcome Trust grant reference 090532/Z/09/Z and Medical Research Council Hub grant G0900747 91070) for generating the sequencing data. Funding to pay the Open Access publication charges for this article was provided by the Wellcome Trust.

## Supplementary Material

Supplementary Data
