# Supplementary material for: Mutations in PIGY: expanding the phenotype of inherited glycosylphosphatidylinositol deficiencies
Source: Hum Mol Genet. 2015 Aug 20;24(21):6146–59. doi: 10.1093/hmg/ddv331 (PMC4599673; doi:10.1093/hmg/ddv331)
Supplement: Supplementary Data [file supp_24_21_6146__index.html]

Mutations in PIGY: expanding the phenotype of inherited glycosylphosphatidylinositol (GPI) deficiencies — Mutations in PIGY: expanding the phenotype of inherited glycosylphosphatidylinositol deficiencies — Mutations in PIGY: expanding the phenotype of inherited glycosylphosphatidylinositol deficiencies — Supplementary Data 

# Mutations in *PIGY*: expanding the phenotype of inherited glycosylphosphatidylinositol deficiencies

## Supplementary Data

Supplementary Data

- Supplementary Data - Pdf file
