# Supplementary material for: Mutations in PIGY: expanding the phenotype of inherited glycosylphosphatidylinositol deficiencies
Source: Hum Mol Genet. 2015 Aug 20;24(21):6146–59. doi: 10.1093/hmg/ddv331 (PMC4599673; doi:10.1093/hmg/ddv331)
Supplement: Supplementary Data [file supp_ddv331_ddv331supp.pdf]

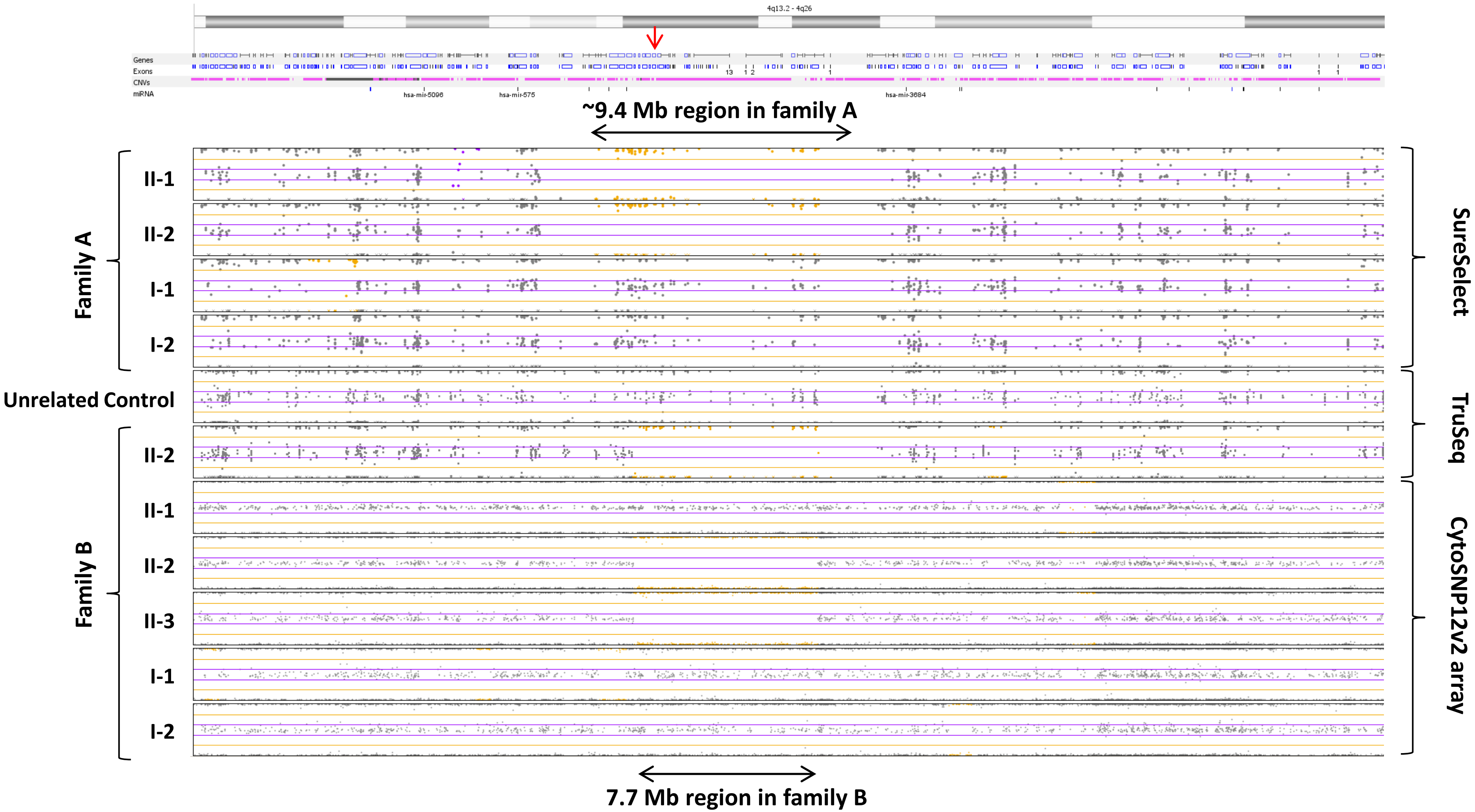

Figure S1: Allelic ratio plots across the *PIGY* locus on chromosome 4. Results are consistent with the homozygous *PIGY* mutations residing within regions of autozygosity, the approximate sizes of which are indicated. For family A, variant calling was performed on all 4 samples, producing a multi-sample VCF. Variants were then filtered out that i) lay in SegDup regions, ii) were indels, iii) did not have a PASS flag, iv) did not have at least 15x coverage in all samples. We then calculated the fraction of reads supporting the variant in relation to the total depth at each locus. This data was then imported as BAFs into Nexus CN and a region of cnLOH was identified at chr4:86,844,948-96,256,674. However, due to the limited probe density, the maximum autozygous region could be as large as 14.8Mb (chr4:84,518,558-99,338,058). These results are consistent with the parents being distantly related. In family B, exome data for individual II-2 was analysed as described above, together with 2 other unrelated samples which had been sequenced in the same batch. One of these unrelated individuals is shown as a control. Results were consistent with the SNP array data which had previously shown a 7.7Mb region of autozygosity shared by both affected siblings (II-2 and II-3). The region shown is chr4:70-120Mb (hg19) and the position of *PIGY* is indicated with a red arrow.

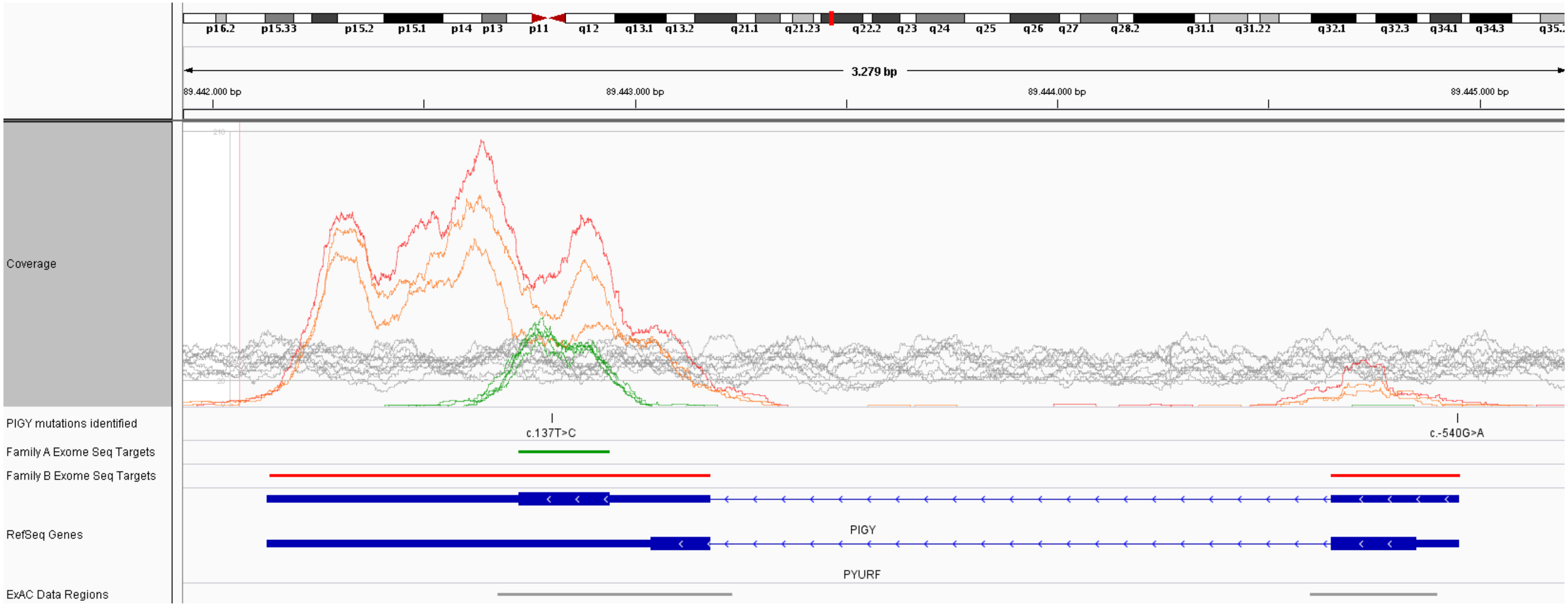

Figure S2: Read coverage analysis of the *PIGY* locus. The top panel shows read coverage of the whole exome sequencing (WES) data for family A (I-1, I-2, II-1, II-2; green lines) and family B (II-2; red line). For comparison, we included data from two unrelated WES datasets that used the same enrichment kit as was used for family B and were run multiplexed on the same sequencing lane (orange lines). Additionally we include coverage data from 10 datasets of an unrelated whole-genome sequencing (WGS) study (grey lines). The horizontal grey line marks 20X coverage. The lower part of the figure shows the locations of the two identified *PIGY* mutations, enrichment kit targets for family A (SureSelect; green bar) and family B (TruSeq; red bars), RefSeq gene annotations (blue) and the considered data regions from the public ExAC database (grey bars). The locus of the coding mutation is targeted and well-covered by all shown WES/WGS datasets/enrichment kits and data describing this locus (e.g., coverage in other WES datasets, allele frequencies, etc.) is available in ExAC as well as in other public databases database. In contrast, the 5'-UTR variant is not targeted (and thus consequently not covered) by the enrichment kit used for family A. Its location near the target boundary of the enrichment kit used for family B explains the low observed coverage which is at the limits of detectability with the employed bioinformatics pipeline (we observed only four reliable reads covering the locus after removing potential PCR duplicates from the data). This means that this variant could have easily been missed with slightly lower overall coverage (cf. the orange coverage curves of the two comparable datasets that contain only one and two reads at this locus respectively) or in a slightly different experimental setting. Moreover, the locus is also uncovered in ExAC and other public WES databases (data not shown) resulting in missing metadata for describing and filtering it. Both loci are well and evenly covered in all shown and over 200 other WGS data sets from a large in-house WGS study that targeted at 25X genome coverage (data not shown) demonstrating the advantage of WGS in situations like this.

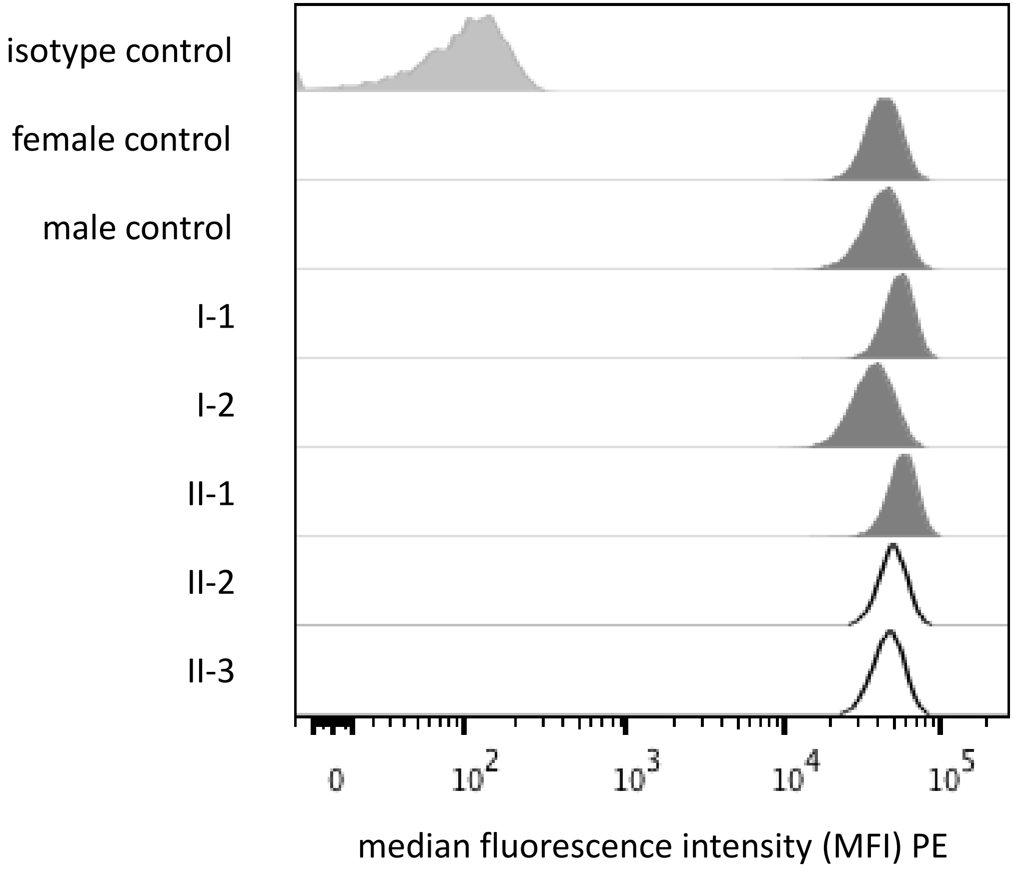

Figure S3: CD16 expression levels in family B measured by flow cytometry. Granulocytes were gated according to FSc and SSc. Shading corresponds to unaffected individuals. No shading corresponds to subjects with developmental delay and microcephaly who are homozygous for c.-540G>A.
